# Novel Therapeutic Options for Solitary Fibrous Tumor: Antiangiogenic Therapy and Beyond

**DOI:** 10.3390/cancers14041064

**Published:** 2022-02-20

**Authors:** Axel de Bernardi, Armelle Dufresne, Florence Mishellany, Jean-Yves Blay, Isabelle Ray-Coquard, Mehdi Brahmi

**Affiliations:** 1 Department of Medical Oncology, Centre Léon Bérard, 28 Rue Laennec, 69008 Lyon, France; debernardi.axel@gmail.com (A.d.B.); armelle.dufresne@lyon.unicancer.fr (A.D.); jean-yves.blay@lyon.unicancer.fr (J.-Y.B.); isabelle.ray-coquard@lyon.unicancer.fr (I.R.-C.); 2 Department of Pathology, Centre Jean Perrin, 58 Rue Montalembert, 63011 Clermont-Ferrand, France; florence.mishellany@clermont.unicancer.fr; 3 Faculty of Medicine, Université Claude Bernard Lyon 1, 69003 Lyon, France

**Keywords:** SFT, rare sarcoma, antiangiogenics, immune-checkpoint inhibitors

## Abstract

**Simple Summary:**

In the latest WHO classification, solitary fibrous tumors (SFTs) are now subdivided into benign SFT (intermediate category (locally aggressive)), SFT NOS (intermediate category (rarely metastasizing)), and malignant SFT. Thanks to recent progress in molecular characterization, the identification of the *NAB2–STAT6* fusion oncogene has emerged as a specific cytogenetic hallmark for SFT. Despite these recent advances in classification and understanding of the molecular pathophysiology of SFT, there are no consensus clinical guidelines regarding systemic treatment. Several new therapeutic options are of interest in this subtype of sarcoma considered as refractory to classical chemotherapy. In case of advanced disease, antiangiogenic therapy might be viewed as the best therapeutic option.

**Abstract:**

SFT is an ultrarare mesenchymal ubiquitous tumor, with an incidence rate <1 case/million people/year. The fifth WHO classification published in April 2020 subdivided SFT into three categories: benign (locally aggressive), NOS (rarely metastasizing), and malignant. Recurrence can occur in up to 10–40% of localized SFTs, and several risk stratification models have been proposed to predict the individual risk of metastatic relapse. The Demicco model is the most widely used and is based on age at presentation, tumor size, and mitotic count. Total en bloc resection is the standard treatment of patients with a localized SFT; in case of advanced disease, the clinical efficacy of conventional chemotherapy remains poor. In this review, we discuss new insights into the biology and the treatment of patients with SFT. *NAB2–STAT6* oncogenic fusion, which is the pathognomonic hallmark of SFT, is supposedly involved in the overexpression of vascular endothelial growth factor (VEGF). These specific biological features encouraged the successful assessment of antiangiogenic drugs. Overall, antiangiogenic therapies showed a significant activity toward SFT in the advanced/metastatic setting. Nevertheless, these promising results warrant additional investigation to be validated, including randomized phase III trials and biological translational analysis, to understand and predict mechanisms of efficacy and resistance. While the therapeutic potential of immunotherapy remains elusive, the use of antiangiogenics as first-line treatment should be considered.

## 1. Introduction

The fifth edition of the World Health Organization (WHO) classification of tumors of soft tissue and bone was published in April 2020, with more than 150 histological subtypes. The classification has been updated with the identification of new distinct molecular subtypes, thanks to the easier access to molecular tools, and these improvements have led to a better understanding of the tumorigenesis of many sarcomas. The classification of conjunctive tumors is crucial, not only for diagnosis and prognostication but also for correct management of patients.

In the fibroblastic and myofibroblastic tumor category, the recognized entity of solitary fibrous tumor (SFT) is described. SFT is a ubiquitous fibroblastic mesenchymal neoplasm originally reported by Klemperer et al. in 1931 [1], with locally invasive properties and a predilection for body cavity sites and serosal membranes; it has only recently been reported in the pleura and/or lung. SFT is known as the “great simulator” of soft-tissue neoplasm due to its many differential diagnoses [2]. The diagnosis of SFT is established by the conjunction of clinical, pathological, immunohistochemical, and molecular features. Identification of the *NAB2* (NGFI-A-binding protein 2)–*STAT6* (signal transduction and activator of transcription 6) fusion oncogene has emerged as a specific cytogenetic hallmark for SFT. Several risk stratification systems have been proposed for SFT to predict which tumors may harbor an aggressive behavior [2,3,4,5]. In the latest WHO classification, SFTs are now subdivided into benign SFT (intermediate category (locally aggressive)), SFT NOS (intermediate category (rarely metastasizing)), and malignant SFT [6].

In this review, we discuss recent progresses in the molecular characterization and therapeutics of SFT and future prospects of tailored therapeutic approaches.

## 2. Description of SFT

### 2.1. Epidemiology

SFT is an ultrarare mesenchymal tumor that represents 3.7% of all soft-tissue sarcomas (STSs) and mesenchymal tumors [7] with a reported incidence rate <1 case/million people/year [8,9]. SFT usually affects adult patients with a peak incidence in the fifth and sixth decades. Pleural SFTs are observed in older patients (median age at diagnosis 56 to 60) compared with intra-abdominal or meningeal SFTs that occur in younger patients (fourth decade) [10].

### 2.2. Clinical and Radiological Presentation

The thorax (pleura, mediastinum, lung parenchyma) is the most common site of initial presentation (30%), but SFT can occur at any extra-thoracic anatomical site [11]. The most frequent extra-pleural location is represented by the head and neck, including meninges (27%), followed by the intra-abdominal region (peritoneum, retroperitoneum, and pelvis) (20%), trunk (10%), and extremities (8%) [12]. SFTs localized in the retroperitoneum, peritoneum, or mediastinum tend to have a more aggressive course compared to other anatomical sites [5,13,14]. SFTs are often slow-growing tumors, with delayed symptom onset. Patients may present with nonspecific pulmonary symptoms such as dyspnea or cough, but often remain asymptomatic despite important tumor volume. Compression of adjacent anatomical structures can be observed for voluminous masses but remains unusual as the median tumor size at diagnosis ranges between 50 and 80 mm [15]. Additionally, few patients (<10%) develop paraneoplastic syndromes which can guide the diagnosis. Hypertrophic osteoarthropathy (Pierre Marie–Bamberger syndrome) is a nonspecific condition rarely associated with pleural SFT. Classical symptoms include distal digital clubbing, periostitis, and synovial effusions, supposedly linked to the paraneoplastic overexpression of vascular endothelial growth factor (VEGF). Fewer than 5% of SFT patients can also present with a refractory hypoglycemic syndrome due to the overproduction of insulin-like growth factor-2 (IGF-2) by large peritoneal or pleural SFTs, called Doege–Potter syndrome [16,17,18].

Consequently, SFTs are frequently diagnosed on incidental radiological findings [19]. Time to diagnosis is classically longer in pleural SFTs compared to extra-pleural SFTs. The radiographic features of SFTs are nonspecific, and computed tomography (CT) usually shows a well-circumscribed isodense mass to skeletal muscle with contrast enhancement in highly vascularized tumors (65%) [15]. SFTs are often characterized by the presence of low-signal-intensity foci on T1- and T2-weighted magnetic resonance imaging (MRI), corresponding to the collagen content. ^18^F-FDG PET/CT may have a limited role in diagnosing SFT in suspected patients, although the presence of multiple high-grade lesions associated with ^18^F-FDG PET hypermetabolism should raise suspicion of a malignant SFT.

### 2.3. Biopathology

#### 2.3.1. Classification

**Figure 1:**
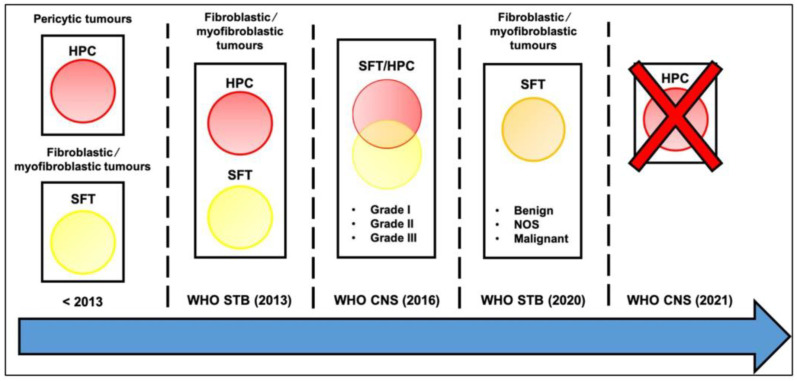
Evolution of the WHO classification of SFTs over time. A crucial update for SFT classification is the development of risk stratification models that resulted in improved prognostication over the traditional benign/malignant distinction (please see the section below).

SFTs belong to the group of fibroblastic and myofibroblastic tumors in the WHO classification [6]. The fifth edition published in April 2020 subdivided SFTs into three categories: benign (locally aggressive), NOS (rarely metastasizing), and malignant [20]. Meningeal SFT, previously known as hemangiopericytoma (HPC), is a rare form of extra-pleural SFT that derives from smooth muscle pericytes surrounding the intraparenchymal microvasculature [21]. Historically, SFTs were considered low-grade tumors contrary to HPCs that displayed aggressive patterns. Despite an apparent distinct clinical behavior, SFTs and HPCs were merged under one disease umbrella in the fourth WHO classification [22]. The fourth revised edition of the WHO Classification of Tumors of CNS published in 2016 introduced the hybrid “SFT/HPC” class, subdivided into three histological grades depending on mitotic count (grade 3 defined by at least five mitoses/10 HPFs) [23]. Most recently, the term of “hemangiopericytoma” was removed from the 2021 WHO Classification of Tumors of CNS to conform with soft-tissue pathology nomenclature [24]. In other words, updated classifications now consider SFTs and HPCs as the same entity at two opposite ends of the same histologic spectrum rather than the strict “benign or malignant” dichotomy that was used for decades (Figure 1).

#### 2.3.2. Prognosis and Risk Stratification

Localized SFTs offer good prognosis after complete surgery. However, recurrence can occur in up to 10–25% of SFTs by 10 years [5,25,26,27]. The risk of metastatic recurrence at 5 years can even rise up to 40% in high-risk patients [13,28,29,30,31,32]. Preferential metastatic sites include lungs, liver, and bone.

SFT recurrence is more frequent in the case of incomplete resection (R1/R2) [25,28], tumor seeding within serosal membranes (pleura, peritoneum), or meningeal or distant hematogenous spread. Small retrospective series tend to show that patients with extra-pleural SFTs have a higher risk of local and metastatic recurrence compared to pleural SFTs [33,34,35]. Meningeal SFTs have a dismal prognosis with frequent local recurrence due to meningeal seeding and early bone metastatic recurrence. Notably, late relapse beyond 10 years and up to 20 years after initial presentation is common, which justifies a long-term follow-up [13,28,29,30,31,32].

Importantly, the development of multivariate risk stratification models has resulted in improved prognostication, such that the traditional benign/malignant distinction is now avoided. Among those models that integrate several clinicopathologic variables to predict the individual risk of metastatic recurrence, the Demicco model (“D-score” or “MDACC score”) is the most widely used in clinical practice and is applicable to SFTs of all extra-meningeal sites [36]. It is based on age at presentation, tumor size, and mitotic count to classify SFTs with a low, moderate, or high risk of developing a metastatic recurrence [37] (Table 1). Another version also includes tumor necrosis. Importantly, dedifferentiation, which corresponds to the abrupt transition from a conventional low-grade SFT component into a high-grade sarcoma and occurs in <1% of primary or recurrent SFTs, remains unpredictable by this model [38].

Ghanim et al. used blood-derived biomarkers to predict event-free-survival (EFS) in intrathoracic SFTs [39]. Elevated preoperative fibrinogen was an independent prognostic marker of poor outcome after curative surgery and associated with malignant SFT. This finding suggests a potential role of innate immune system overactivation in SFT prognosis. 

The presence of *TP53* and/or *TERT* mutations seems to be correlated with malignant SFTs associated with an aggressive biological behavior. 

#### 2.3.3. Tumorigenesis, Pathology, and Molecular Alterations

**Figure 2:**
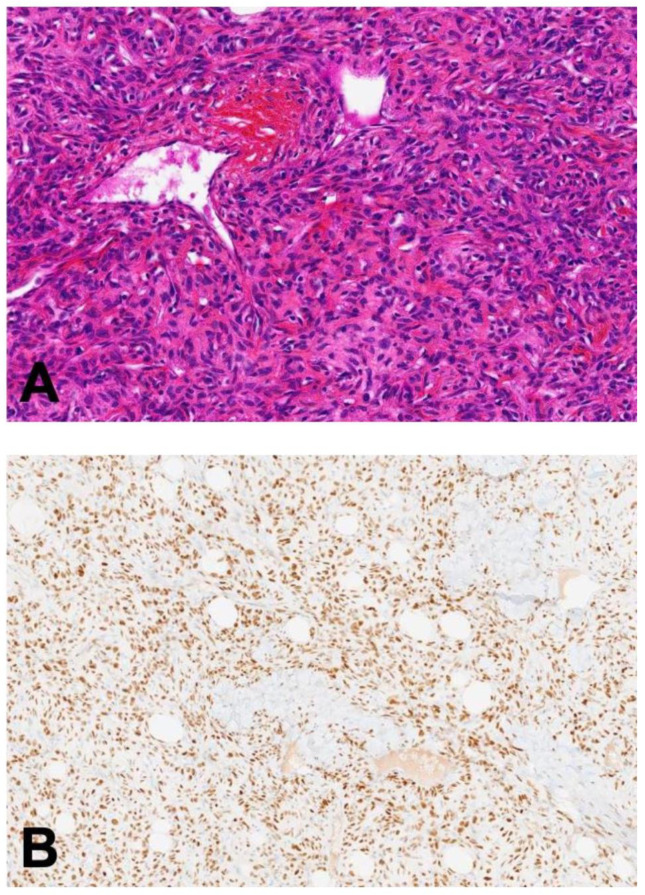
(**A**) Morphological appearance of SFT composed of spindle cells with a patternless architecture and a dense hyalinized collagenous stroma. Magnification: ×40; staining: hematoxylin, eosin, and Safran (HES). (**B**) Diffuse and strong STAT6 nuclear staining in SFT tumor cells. Magnification: ×40; staining: STAT6 antibody.

The macroscopic appearance of SFT is a well-defined, lobulated, firm mass surrounded by a serosal pseudo-capsule. The wide histological spectrum of SFT ranges from morphologically paucicellular to highly cellular tumors. SFTs are composed of atypical spindle cells with fusiform nuclei arranged haphazardly with a suggestive “patternless pattern”, surrounded by a dense stromal collagen with thin collagen bands (Figure 2A) and admixed with a characteristic branching staghorn (hemangiopericytoma-like) vasculature. Hypocellular phenotypes have a low mitotic count, and nuclear pleomorphism or necrosis is classically absent. These histopathologic patterns are not specific to SFTs and can also be observed in other mesenchymal tumors [40,41].

In practice, the detection of the *NAB2–STAT6* fusion gene (detailed below) with cytogenetic methods such as fluorescent in situ hybridization (FISH) is impossible due to the small size of the inverted sequence and the proximity of the *NAB2* and *STAT6* loci. For routine diagnosis, STAT6 is a robust immunohistochemical surrogate marker of all *NAB2–STAT6* fusion transcripts. A strong nuclear expression of the C-terminal part of STAT6 has good diagnostic performance with excellent sensitivity (98%) and specificity (>85%) [42,43,44,45,46] (Figure 2B). PCR-based detection of STAT6 is less sensitive for diagnosis due to the diversity of possible breakpoints in fusion transcripts. However, STAT6 is inconsistently expressed and might be absent in some SFT cases. STAT6 is rarely overexpressed in the nucleus and cytoplasm of well-differentiated (WD-LS) and dedifferentiated liposarcoma (DD-LPS) cells [47]. If that differential diagnosis is discussed, a complementary analysis of MDM2 and CDK4 expression in IHC can be useful as they are overexpressed in WD-LS/DD-LPS but not in SFT. Other standard IHC markers can be used in combination (CD34, Bcl2, CD99, vimentin, desmin, S100 protein, epithelial markers) with good sensitivity but a low specificity. Lastly, GRIA2 and ALDH1 are under investigation [48,49].

The WHO classification updates were justified by the discovery of a shared cytogenetic signature across all anatomical sites in 2013 by three different research groups: the *NAB2–STAT6* fusion oncogene [41,50,51,52]. The *NAB2–STAT6* fusion gene is detected in nearly all SFT cases, which suggests a driver role in tumor development. *NAB2* and *STAT6* are adjacent genes transcribed in opposite directions at the locus 12q13 [41,51,52].

NAB2 is a transcriptional repressor that binds to the inhibitory domain of *EGR-1* (early growth response 1) through its N-terminal binding domain (NAB2 conserved domain 1—NCD1) to have transcriptional control over EGR-1 target genes, including IGF-2 or FGFR1. *EGR-1* is a downstream gene of the MAPK/ERK pathway [53]. EGR-1 enhances the transcription of cell-cycle regulatory proteins such as cyclin D1 that promotes tumor cell proliferation, which in turns activates the MAPK/ERK signaling pathway and auto-activates the expression of EGR-1 through a positive feedback loop. Additionally, the transcription of *EGR-1* is upregulated by various growth factors such as IGF-1 and its receptor IGFR-1. *EGR-1* also plays a role in the systemic dissemination of tumor cells. Indeed, epithelial–mesenchymal transition (EMT) was reported to be triggered by the *EGR-1*-induced upregulation of Slug and Snail via the ERK1/2 and PI3K/Akt pathways in ovarian cancer cells [54]. Proangiogenic growth factors such as basic fibroblast growth factor (bFGF) and VEGF-A are EGR-1 target genes that contribute to tumor angiogenesis. EGR-1 can directly activate the transcription of these factors [55] or be stimulated via the ERK1/2 pathway after hypoxia-inducible factor (HIF)-1α overexpression in response to hypoxia in the tumoral microenvironment.

STAT6 (signal transducer and activator of transcription 6) is a transcription factor usually involved in allergic and immune signaling pathways with a role in tumorigenesis [56,57]. The Src homology 2 domain (SH2) is crucial to bind to IL-4R or IL-13R and trigger STAT6 phosphorylation by Janus (JAK) and TyK2 kinases [58]. In its dimerized form, STAT6 is activated, whereby it can enter the nucleus and bind to DNA promoters through its DNA-binding domain (DBD1).

**Figure 3:**
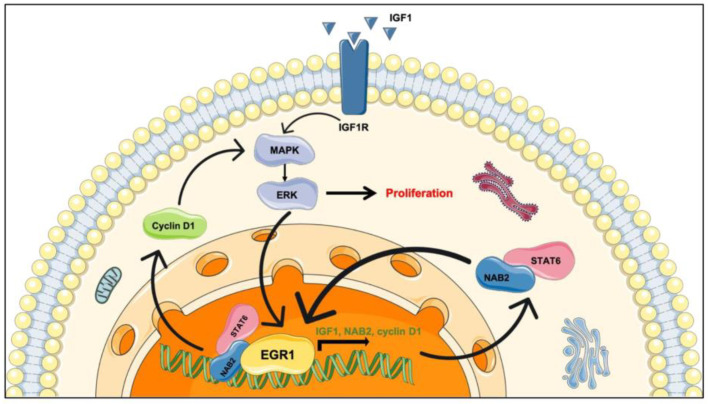
Suspected role of the NAB2–STAT6 fusion transcript in SFT tumorigenesis.

In SFT, the recurrent intrachromosomal inversion in the long arm of chromosome 12 leads to the replacement of at least one of the three repressor domains of *NAB2* (NCD1, NCD2, CID) by the transactivation domain (TAD) of *STAT6*. *NAB2* and *STAT6* are fused in a common direction of transcription, which results in the transcription of a chimeric NAB2–STAT6 fusion protein from the *NAB2* promoter [50,52]. Then, the fusion transcript translocates to the nucleus and constitutively activates EGR-1-responsive genes [41] (Figure 3). Intriguingly, *NAB2* and *EGR-1* are mutual targets to each other, which creates a positive feedback loop and strengthens the abnormal accumulation of the *NAB2–STAT6* fusion transcript in the nucleus of SFT cells. This specificity is a hallmark of SFT used for differential diagnosis with other tumors.

Barthelmess and colleagues first suggested that the *NAB2 exon4–STAT6 exon2* (N4S2) and the *NAB2 exon6–STAT6 exon16/17* (N6S16/17) fusion variants might be associated with distinct clinical features. *N4S2* was mostly found in older patients with less aggressive SFTs and deep extra-thoracic lesions. On the other hand, *N6S16/17* was more frequent in younger patients with aggressive phenotypes and clinical behavior usually found in meningeal SFTs [50]. Since then, other studies failed to demonstrate a clear impact of fusion variants on prognosis, probably due to a short follow-up that did not take into account late recurrences [59,60,61,62,63]. In a recent retrospective cohort with long-term follow-up, Georgiesh et al. investigated the clinicopathological and prognostic impact of the STAT6-Full (intact STAT6 domains) and STAT6-TAD (contains only the STAT6 TAD domain) variants by RNA sequencing [64]. A total of 39 patients with localized extra-meningeal SFTs were enrolled. Patients with STAT6-TAD tumors had a worse prognosis, with a higher mitotic count and a 10 year recurrence-free survival rate of 25% (vs. 78% for STAT6-Full patients). These promising results need further confirmation in prospective trials to conclude on their prognostic value.

## 3. Therapeutic Options for SFT

Patients with SFTs should be managed within sarcoma reference centers, by a dedicated multidisciplinary team with a pathologist, radiologist, surgical oncologist, radiation oncologist, and medical oncologist who are familiar with the nuances of this disease [65,66,67,68]. Each case has to be discussed in a specialized multidisciplinary tumor board (MTB) to determine the best individualized therapeutic strategy.

For response assessment, the use of both Response Evaluation Criteria in Solid Tumors (RECIST) and the new Choi criteria (i.e., a 10% decrease in tumor size or a more than 15% decrease in tumor density) might be of interest to evaluate therapeutic response to antiangiogenic agents [69,70,71]. Choi criteria were originally developed to predict the response of advanced gastrointestinal stromal tumor (GIST) to imatinib. Due to the hypervascularized nature of SFT, recent studies used both Choi and RECIST criteria to assess radiological response. The retrospective studies detailed below reported few responses with RECIST criteria but 46% to 79% response with Choi criteria [72,73,74,75,76]. However, the predictive value of Choi criteria in SFTs should be interpreted cautiously. There are no data on the use of Choi criteria in patients with advanced SFTs treated with chemotherapy.

### 3.1. Localized Disease

#### 3.1.1. Surgery

Complete en bloc surgical resection with negative margins (R0) is the gold-standard treatment for localized disease [77]. The 10 year overall survival (OS) in patients with SFTs resected with negative margins ranges from 54% to 89% [78,79,80]. In pleural SFTs, the modalities of resection include wedge resection, lobectomy, or pneumonectomy, associated with chest wall or diaphragm resection if necessary [77,81,82]. In their retrospective series, Lahon et al. resected localized pleural SFTs in 157 patients. Despite R0 margins, 15 patients (10%) recurred with a median time to recurrence of 29 months (10 local and five metastatic recurrences) [81]. Re-resection of local recurrence could achieve local control in 9/10 patients. The 5 year and 10 year OS rates were 86% and 77%, respectively. Similarly, Lococo et al. reported disease recurrence in 15/50 patients (six local and nine metastatic recurrences) with localized pleural SFTs resected with negative margins, yielding 5 year and 10 year OS rates of 81% and 67%, respectively [83]. Complete surgical resection is also the primary approach in extra-pleural tumors, with procedures paralleling other sarcoma surgeries from similar anatomical sites.

#### 3.1.2. Radiation Therapy

Several strategies can be discussed in the postoperative setting, depending on surgical margins and risk stratification models. Despite limited available prospective data, multiple observational studies report improved local control in high-risk SFT patients treated with adjuvant radiotherapy (RT). Nevertheless, there is no clear demonstration of an OS benefit of adjuvant RT [13,14,26,33,34,84,85,86,87,88]. In 2020, Haas et al. investigated the role of perioperative RT in localized extra-meningeal SFT [14]. In this cohort, 428/549 patients (78%) received surgery alone and 121 (22%) were treated with surgery and RT. Overall, 48% of patients (*n* = 58) received surgery followed by adjuvant RT, with a lower risk of local progression (*p* = 0.012), and a 96% local control rate at 5 years. However, this association did not translate into an OS benefit (*p* = 0.325). Preoperative RT was assessed in the STRASS phase III randomized trial, which included intra-abdominal sarcomas of different histotypes, but SFT cases were underrepresented, leading to inconclusive results in that population [88,89]. An international retrospective observational study performed across seven specialized sarcoma centers aimed to better define the benefit of definitive RT in this disease [90]. Forty patients with locally advanced or metastatic SFT (nine pleural, 16 soft-tissue, 10 meningeal, three head and neck, and two other SFTs) were treated with definitive RT, receiving approximately 60 Gy, with an objective response rate of 67%. At 5 years, the local control (LC) rate was 81.3%, and the OS rate was 87.5%. In case of palliative RT (39 Gy in conventional fractionation), the overall response rate (ORR) was 38%, and the 5 year LC and OS rates were 62.5% and 54.2% respectively. Therefore, radiation doses can range from 39 Gy (in conventional fractions) for palliative care to 60 Gy to achieve durable control.

In summary, observation is recommended in patients with negative margins (R0) without high-risk histologic features. In the case of intermediate- to high-risk SFT with positive margins (R1/R2), re-resection should be discussed for fit patients if complete resection can be achieved with minimal morbidity. If the patient is unfit for further resection or R0 surgery cannot be technically achieved (due to anatomical site), then adjuvant RT is a reasonable option. Surgeons may adopt a conservative attitude in selected cases to preserve organ functions when postoperative RT can be delivered, given the favorable long-term outcome. Adjuvant RT should systematically be considered for high-risk SFTs, such as malignant SFTs of the central nervous system (CNS), considering the very high risk of local recurrence. Neoadjuvant RT can be an option in selected cases to improve tumor resectability or when wound complications are predicted to be manageable.

#### 3.1.3. (Neo)Adjuvant Chemotherapy

In patients with localized, resectable SFTs, there is no evidence supporting the use of systemic therapies in the (neo)adjuvant setting [18]. A review of the literature shows only limited data on this subject and consists mostly of a few case reports [91,92,93]. Even if the relevance of adjuvant chemotherapy (CT) in resected SFTs is still unknown due to a lack of data, it is important to note that SFTs marginally benefit from traditional sarcoma CT with low response rates (Table 2). Importantly, adjuvant CT should never be intended to rescue inadequate surgery. Nevertheless, for some patients with high-risk SFTs and/or large malignant tumors, the use of neoadjuvant CT should be discussed at a specialized MTB. Importantly, eligible patients should be managed within clinical studies. Therefore, in the case of locally advanced tumors, if R0 surgery is not feasible apart from mutilating surgery, neoadjuvant CT is an option. On the basis of the response rate data (Table 2), an anthracycline-based regimen (plus ifosfamide or dacarbazine) can be considered as the regimen of choice. Importantly, early tumor response assessment is required to avoid delaying surgery in the case of nonresponding disease.

### 3.2. Advanced and Metastatic Disease

#### 3.2.1. Surgery, Ablations, or RT

Metachronous (disease-free interval >1 year), resectable lung metastases without extrapulmonary disease may be managed with surgery as standard treatment applied to sarcoma, if complete excision of all lesions is feasible. Surgery, ablations, or RT of extrapulmonary metastases may also be an option in highly selected cases [65,66,67].

#### 3.2.2. Chemotherapy

In cases of synchronous and/or unresectable lung metastases and in cases of extrapulmonary metastatic disease, patients are candidates for systemic treatment, even though a standard medical approach is currently not established. Published data on the response of SFTs to conventional chemotherapy are limited, and results gathering small case series, retrospective studies, and predictive preclinical models show conflicting results. Table 2 summarizes the data available on the efficacy of systemic agents in SFT. 

For anthracycline-based therapies, ORR ranges between 0% and 20%, and stable disease (SD) is achieved in 26–65% of cases with a median PFS (mPFS) and median OS (mOS) of 4–5.2 months and 11.5–14.6 months, respectively [72,74,94,95,99]. Ifosfamide monotherapy as first-line treatment was also assessed with a 10% ORR and 26% SD. Dacarbazine monotherapy showed a 37.5% ORR and an mPFS of 7 months [96]. Despite a 7.9–9.1% ORR, trabectedin is an important alternative in subsequent lines, as disease stabilization can be achieved in 42–73% of cases with an mPFS of 3.7–11.6 months and an mOS of 14.3–22.3 months [97,98,110]. Consequently, the use of trabectedin is often associated with a meaningful clinical benefit in patients with advanced SFTs. Subsequent lines of chemotherapy consisted of cytotoxic agents (in monotherapy or in combination) used routinely for STS treatment, but showed no objective response.

Common and frequent chemotherapy-induced adverse events such as high-grade mucositis, nausea and vomiting, or hematologic toxicities were reported with all the regimens. Additionally, patients could experience congestive heart failure (associated or not with a reduction in cardiac ejection fraction) with anthracycline, ifosfamide-induced encephalopathy or hepatotoxicity, and rhabdomyolysis with trabectedin. The toxicity profiles were similar to those classically observed in STS.

Overall, the consistent response rates observed across retrospective series tend to confirm the limited efficacy of conventional chemotherapy in advanced SFT. The STRADA randomized phase II study (NCT03023124) will prospectively compare trabectedin (1.3–1.5 mg/m^2^) to doxorubicin (75 mg/m^2^) plus dacarbazine (400 mg/m^2^/day, days 1, 2) in 50 patients with advanced SFTs. The Italian phase II single-arm ERASING trial (NCT03840772) will also evaluate the activity of eribulin in that population. Interestingly, the Choi response rate is one of the secondary outcomes of the ERASING and STRADA trials, which will provide data in these populations.

In summary, even if there is no formal gold standard, patients with metastatic SFTs are treated routinely in the same way as other STSs, using anthracycline as first-line therapy, while ifosfamide, dacarbazine, and trabectedin are options for second line and beyond. Best supportive care alone is an alternative for unfit patients, regarding the toxicities of those drugs. Otherwise, there is no demonstration that multiagent CT improves patient survival, and single-agent chemotherapy remains the standard.

#### 3.2.3. Antiangiogenic Therapies and Other Targeted Therapies

SFTs are highly vascularized tumors with high expression rates of proteins involved in angiogenic pathways such as platelet-derived growth factor receptor (PDGFR) and vascular endothelial growth factor receptor (VEGFR) [111]. Angiogenesis is an important feature involved in tumor growth and metastatic diffusion of SFT. Accordingly, the inhibition of angiogenesis pathways was suspected to be a key therapeutic target to inhibit tumor cell proliferation. Considering the angiogenic properties of SFTs, several case reports and clinical trials investigated various inhibitors of angiogenesis with a promising activity in SFTs.

Prolonged disease control up to 30 months has been reported with antiangiogenic agents (sunitinib, sorafenib, pazopanib, temozolomide–bevacizumab) in case reports, retrospective studies, and phase I and phase II trials [76,95,101,102,103,104,105,106]. ORR varied from 0% to 79% with an mPFS ranging between 4.7 months and 9.7 months using Choi criteria. Antiangiogenic agents were positioned in subsequent lines after chemotherapy resistance.

A prospective single-arm phase II trial confirmed the efficacy of pazopanib in two cohorts of typical (*n* = 34) and malignant/dedifferentiated (DD) SFTs (*n* = 36) with 58% and 51% partial response (PR), respectively, according to Choi criteria. [108,109]. Interestingly, pazopanib was associated with a very high clinical benefit rate in the typical SFT cohort (97%). The outcomes of PFS were also more favorable in the typical SFT cohort than in the malignant/DD SFT cohort, with an mPFS of 9.8 month using Choi criteria and 12.1 months based on RECIST criteria in the typical SFT cohort, versus 5.6 months for the malignant/DD SFT group. Notably, the inclusion of patients with DD-SFT was stopped due to cases of hyper-progression under pazopanib in a planned interim analysis. The toxicity profiles were similar between the two cohorts and consistent with those reported in previous clinical trials.

Importantly, pazopanib harbors a multifaceted cardiovascular toxicity profile including cardiomyopathy, QTc-interval prolongation, and hypertension. Therefore, a cardiologic workup should always be proposed in patients with a preexisting cardiovascular condition. Even in the absence of comorbidity, it should be considered in patients previously treated with anthracycline-based CT.

In summary, according to the aforementioned results, pazopanib can be a treatment option in the first-line setting for typical SFT.

#### 3.2.4. IGF-1 Inhibitors

IGF-1 is overexpressed in SFT, and treatment regimens using figitumumab, a fully human IgG2 anti-IGF-1 (IGF-1R) monoclonal antibody, demonstrated tumor responses in a few patients with advanced SFTs [106,112]. In a phase I trial assessing the combination of figitumumab and the mTOR inhibitor everolimus, the only PR (among 18 evaluable patients) was observed in a patient with SFT [112]. Unfortunately, Pfizer ceased the development of the drug in 2011 and has stopped its manufacture.

#### 3.2.5. Immunotherapy

Immunotherapy is another promising approach for SFT. Available data on the SFT immune microenvironment mainly come from retrospective studies.

Tazzari et al. first reported an immunosuppressed environment at the tumor site marked by the absence of a granulocytic MDSC (gMDSC) infiltrate [113]. Samples treated with sunitinib malate were enriched in activated CD8^+^ and CD4^+^ cells, which suggests that antiangiogenic therapies might modulate the T-cell immune infiltrate in SFTs. A translational study on 16 intracranial SFT/HPC specimens (13 HPCs, three SFTs) evaluated the correlation of PD-1, PD-L1, and tumor-infiltrating lymphocyte (TIL) expression with prognosis [114]. PD-L1 was expressed in all tumors. As an individual biomarker, a diffuse or intense PD-L1 staining was associated with a shorter time to treatment failure (TTF). Diffuse PD-L1 staining coupled with the absence of CD8 expression was significantly associated with a shorter TTF (*p* = 0.005). Consequently, the conjunction of diffuse PD-L1 IHC staining and the absence of CD8 expression may predict the early occurrence of extracranial metastases in SFTs [114]. Dancsok et al. systematically evaluated the expression of immune checkpoint biomarkers and TILs in a variety of sarcoma subtypes [115]. Among the 16 SFT cases included in the study, PD-1 and PD-L1 were infrequently expressed with sparse TILs. On the basis of the results in the entire cohort, the authors suggested that T-cell immune infiltrate might be less frequent in translocation-associated sarcomas, such as SFTs. More recently, Berghoff et al. investigated the inflammatory tumor microenvironment in 74 specimens of meningeal tumors including 12 cases (16.2%) of HPC and seven cases (9.5%) of meningeal SFT [116]. TILs were present in all SFT cases and 11/12 cases (91.7%) of HPC. PD-L1 was only expressed in 1/12 (8.3%) cases of HPC.

Several cases of partial response to anti-PD1 or -PDL1 therapies have been reported in the recent literature. Boothe et al. published the case of a patient with advanced malignant pleural SFT who experienced a long-lasting near-complete response after 31 cycles of pembrolizumab. PD-L1 was positive in IHC (5%), and a mutation in exon 9 of *MLH1* (E234Q) was detected after next-generation sequencing (NGS) [117]. Lastly, in a phase II trial of pembrolizumab in sarcomas, one case of SFT was the only PR reported in the study [118]. Given the suspected role of the immune system in these neoplasms, a phase III trial is currently ongoing to compare nivolumab + ipililumab to pazopanib in adults with advanced rare STSs including SFTs (NCT04741438).

## 4. Conclusions

Sarcomas represent a highly heterogeneous group of tumors, both in clinical and in genomic settings; thus, they should be treated separately. Furthermore, recent clinical trials exploring unselected sarcoma histotype populations [119] failed to improve patient outcome. Therefore, each subtype should still be treated separately.

SFTs are poorly sensitive to conventional chemotherapy. Nevertheless, the pathognomonic *NAB2–STAT6* oncogenic fusion that induces IGF-1 overexpression and angiogenesis in the tumor microenvironment might help considering SFT as a targetable sarcoma. Those biological insights recently translated into clinical management, especially IGF-1 inhibitor (figitumumab) and antiangiogenic drugs (including pazopanib, sunitinib and sorafenib). The results presented in this review suggest that antiangiogenic therapies such as pazopanib could be of interest for first-line treatment, while data on the efficacy of immunotherapy remain scarce, and more results are needed.

Future approaches in advanced SFT treatments should focus on international collaborations to develop large, randomized phase III trials to assess the efficacy of antiangiogenics and/or immune checkpoint inhibitors (ICI) (PD-1 inhibitors and PD-L1 inhibitors) compared to conventional chemotherapy. Additionally, a randomized phase III trial comparing antiangiogenic therapy alone versus antiangiogenic therapy plus anti-PD-1/PD-L1, as well as ICI in combination, would be of interest. For localized disease, could (neo)adjuvant therapy based on antiangiogenics and/or ICI decrease recurrence rate in high-risk patients? Nevertheless, an international approach to research on this rare disease is unavoidable, and additional biological ancillary studies are highly recommended.

In conclusion, SFT is a rare STS subtype for which standard chemotherapy has been reported to have limited efficacy. Overall, our review underlines the modest activity of standard chemotherapy in SFT but confirms that antiangiogenic agents have interesting activity and might be considered as the best therapeutic option in the advanced setting. However, the prognosis remains poor, and the inclusion of patients in clinical trials is highly recommended.

## Figures and Tables

**Table 1:** Risk stratification model proposed by Demicco et al. [2].

Risk Factor	Cut-Off	Points Assigned	
		3-Variable Model	4-Variable Model
Patient age (years)	<55	0	0
	>55	1	1
Mitoses/mm^2^	0	0	0
	0.5–1.5	1	1
	≥2	2	2
Tumor size (cm)	0–4.9	0	0
	5–9.9	1	1
	10–14.9	2	2
	≥15	3	3
Tumor necrosis	<10%	N/A	0
	≥10%	N/A	1
Risk	Low	0–2 points	0–3 points
	Intermediate	3–4 points	4–5 points
	High	5–6 points	6–7 points

**Table 2:** Summary of available data on systemic agents in SFT.

Authors	Design	*n*	Drug (*n*)	Response/Duration/RR	mPFS (Months)	mOS (Months)
Constantinidou et al. 2012 [94]	R	24	Anthracycline-based (75 mg/m^2^) (*n* = 17) Non-anthracycline-based (*n* = 7)	– 1 PR, 40% SD, 50% PD – 5 SD, 2 PD	4.2 (95% CI: 0–10.1)	14.6 (95% CI: 9.3–19.9)
Levard et al. 2013 [95]	R	23	Doxorubicin alone (*n* = 9) Pegylated liposomal doxorubicin (*n* = 1) Doxorubicin + ifosfamide (*n* = 8) Doxorubicin + palifosfamide (*n* = 1). Vinorelbine (*n* = 1) Paclitaxel (*n* = 1) Carboplatin and paclitaxel (*n* = 1) Brostallicin (*n* = 1)	2 PR (8.7%) in doxorubicin-based, 13 SD (59%), 8 PD (35%). 9 pts (39%) progression-free at 6 months	5.2 (95% CI: 3.2–7.1)	33.5 (95% CI: 14.2–52.8) (whole cohort, *n* = 30)
Stacchiotti et al. 2013 [74]	R	31	Anthracycline monotherapy (*n* = 8), Anthracycline + ifosfamide (*n* = 23) Ifosfamide (*n* = 19)	– RECIST: 6 PR (20%), 8 SD (27%), 16 PD (53%). 20% progression-free at 6 months. – 2 PR (10%), 5 SD (26%), 12 PD (63%)	4 (range 2–15)	11.5 (range 3–50)
Stacchiotti et al. 2013 [96]	R	8	Dacarbazine monotherapy (*n* = 8), (1200 mg/m^2^)	RECIST: 3 PR, 4SD, 1PD	7 (range 2–12)	-
Park et al. 2013 [72]	R	21	Doxorubicin-based (*n* = 15) Gemcitabine-based (*n* = 5) Paclitaxel (*n* = 5)	0 PR, 16 SD (89%), 2 PD (11%). 5 (28%) progression-free >6 months	4.6 (95% CI: 4.0–5.3)	10.3 years (95% CI: 5.7– 14.9 years)
Khalifa et al. 2015 [97]	R	11	Trabectedin (1.5 mg/m^2^)	RECIST: 1 PR (9.1%), 8 SD (72.7%)	11.6 (95 % CI = 2.0; 15.2)	22.3 (95 % CI = 9.1; NR)
Le Cesne et al. 2015 [98]	R	13	Trabectedin 1.5 mg/m^2^	-	7.6 (95% CI: 1.6–13.7)	14.3 (95% CI: 0.8–27.8)
Schöffski et al. 2020 [99]	R	26	Doxorubicin (*n* = 15) (57.7%) Doxorubicin + ifosfamide (*n* = 3) (11.5%) Doxorubicin/olaratumab (*n* = 2) (7.7%) Doxorubicin + evofosfamide (*n* = 1) Doxorubicin + ifosfamide + cisplatin (*n* = 1) *2nd line*: ifosfamide (*n* = 5), pazopanib (*n* = 5)	Doxorubicin-based: 2 PR (13.3%), 4 SD (26.7%) Ifosfamide: no response Pazopanib: 3 PR (60%)	34.1 (95% CI: 1.0–157.1)	56.0 (95% CI: 0.3–258.3)
Outani et al. 2020 [100]	R	31	Anthracycline-based (*n* = 11) Eribulin mesylate (*n* = 4) Gemcitabine + docetaxel (*n* = 10) Ifosfamide-based (*n* = 7) Pazopanib (*n* = 22) Trabectedin (*n* = 6) Other (*n* = 13) Multidrug regimen (*n* = 21)	-	-	55 (95% CI: 40–86)
Kobayashi et al. 2020	R	140	Trabectedin (1.2 mg/m^2^)	11 PR (7.9%), 54 SD (41.9%) with 25/54 SD >6 months	3.7 (95% CI: 2.8–5.7)	16.4 (95% CI: 11.5–21.2)
Mulamalla et al. 2008 [101]	CR	1	Sunitinib	SD for 6 months	-	-
De Pas et al. 2008 [102]	CR	1	Imatinib	PR for 21 months with major clinical benefit	-	-
George et al. 2009 [103]	R	48	Sunitinib (37.5 mg)	1 PR, 11 SD + PR (22%) at 16 weeks, 7 (14%) at 24 weeks.	1.8	-
Domont et al. 2009 [104]	P	2	Dacarbazine (1000 mg/m^2^) + sorafenib (400 mg) Sunitinib (50 mg)	PR, PD at 1.5 year SD > 6 months	-	-
Park et al. 2011 [73]	R	14	Temozolomide (150 mg/m^2^)–bevacizumab (5 mg/kg)	Choi: 11 PR (79%), 2 SD	9.7 (95% CI: 7.31–not estimable)	-
Valentin et al. 2013 [105]	P	5	Sorafenib (800 mg)	No objective response, 2/5 SD (9 months)	-	19.7
Levard et al. 2013 [95]	R	10	Pazopanib (800 mg) (*n* = 6) Sunitinib (37.5 mg) (*n* = 4)	No objective response, 5 SD, 5 PD 4 pts (40%) progression-free >6 months: at 8.0 and 14.0 (pazopanib), and 29.5 and 29.9 months (sunitinib).	5.1 (95% CI: 0.7–9.6).	-
Stacchiotti et al. 2010 [106]	R	11	Sunitinib (37.5 mg)	6 PR, 1 SD (Choi)Response >6 months in 5 pts	-	-
Stacchiotti et al. 2014 [107]	R	6	Pazopanib (800 mg)	No RECIST response 1 PR, 2 SD (Choi)	3 (range 1–15)	-
Maruzzo et al. 2015 [76]	P	13	Pazopanib 1st line (800 mg)	5 PR (46%), 4 SD (36%) (Choi)	4.7 (95% CI: 4.8–7.4)	13.3 (95% CI: 3.9–22.6)
Martin-Broto et al. 2019 [108]	P	36	Pazopanib (800 mg)	Choi: 18 PR (51%), 9 SD (26%) RECIST: 2 PR (6%), 21 SD (60%)	5.6 (95% CI: 4.51–6.62)	NR
Martin-Broto et al. 2020 [109]	P	31	Pazopanib (800 mg)	18 PR (58%), 12 SD (39%), 1 PD (3%)	12.1 (range 2.6–21.7)	49.8 (range 8.2–91.3)

(R = retrospective study; P = prospective study; CR = case report; pts = patients; CI = confidence interval; NR = not reached).
